# Subcutaneous emphysema of the leg after hardware removal and bone allografting for infected non-union of the distal femur

**DOI:** 10.1186/s12891-017-1706-y

**Published:** 2017-08-15

**Authors:** Vincenzo Denaro, Umile Giuseppe Longo, Giuseppe Salvatore, Vincenzo Candela, Nicola Maffulli

**Affiliations:** 1 Department of Orthopaedic and Trauma Surgery, Campus Bio-Medico University, Via Alvaro del Portillo, 200, 00128 Trigoria, Rome Italy; 2 Centre for Sports and Exercise Medicine, Barts and The London School of Medicine and Dentistry, Mile End Hospital, 275 Bancroft Road, London, E1 4DG UK; 3 University of Salerno School of Medicine, Salerno, Italy

**Keywords:** Gangrene, Fracture, Hardware removal, Femur

## Abstract

**Background:**

Infected non-unions of the supracondylar region of the femur are uncommon. Even though hardware removal is a common procedure, it may lead to complications, including neurovascular injury, refracture, worsening pain or recurrence of deformity.

**Case presentation:**

We report on a male who developed subcutaneous emphysema of the leg after hardware removal and bone allografting for an infected non-union of the distal femur. He was managed by debridement of the surgical wound, antibiotic therapy, multiple fasciotomies, and application of a VAC (vacuum-assisted closure) system.

**Conclusions:**

Although subcutaneous emphysema of the leg after hardware removal and bone allografting for infected non-union of the distal femur is extremely rare, the potential life treating complications and their potential impact on the functional status of the patient have to be taken into consideration when counseling patients about this procedure. Even when it is not possible to identify a bacterial pathogen responsible for the subcutaneous emphysema of the leg, prompt intervention may save the limb of the patient.

## Background

Infected non-unions are defined as a state of failure of union and persistence of infection at the fracture site for 6 to 8 months [1, 2]. Infected non-unions of the supracondylar region of the femur are uncommon [1–3]. However, when they occur, management is challenging. They usually originate after severe open fractures with extensive comminution and segmental bone loss or after internal fixation of a comminuted closed fracture [1, 4]. Factors which complicate management and recovery include osteomyelitis, polybacterial multidrug-resistant infection, osteopenia, soft-tissue loss with multiple sinuses, complex deformities with limb-length inequality, and stiffness of the adjacent joint [5].

Often patients require hardware removal and further orthopaedic interventions. Even though hardware removal is a common procedure, it may lead to complications, including neurovascular injury, refracture, worsening pain or recurrence of deformity [6, 7].

We report on a male who developed subcutaneous emphysema of the leg after hardware removal and bone allografting for an infected non-union of the distal femur. He was managed by debridement of the surgical wound, antibiotic therapy, multiple fasciotomies, and application of a VAC (vacuum-assisted closure) system.

To the best of our knowledge, this is the first reported incidence of subcutaneous emphysema of the leg after hardware removal and bone allografting for infected non-union of the distal femur. Our patient was informed that data concerning the case would be submitted for publication, and gave written consent.

## Case presentation

**Fig. 1:**
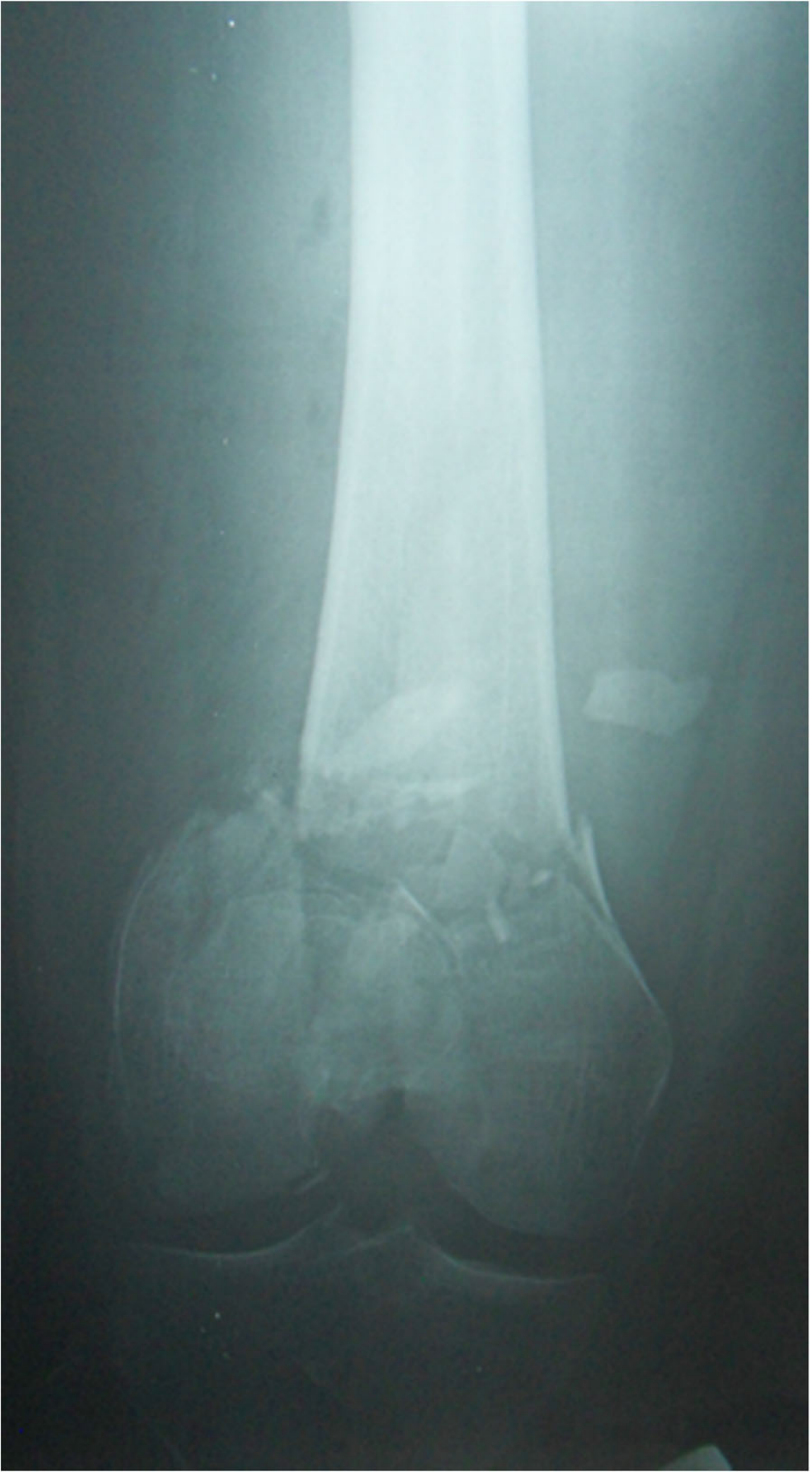
Anteroposterior radiograph of the right supra-intercondylar multifragmentary fracture of the distal femur

**Fig. 2:**
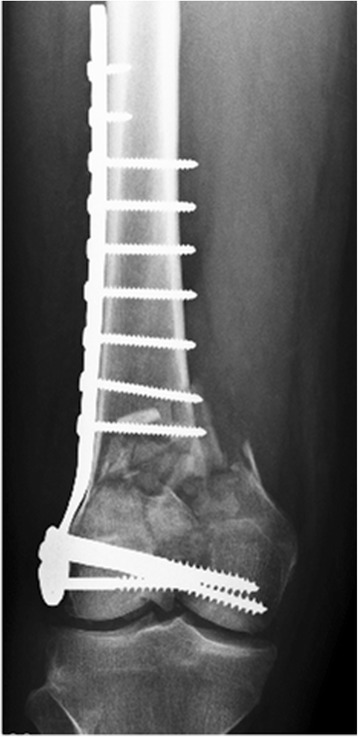
Anteroposterior radiograph of the right femur 3 days post-operatively

A 59-year-old male sustained an open right supra-intercondylar fracture of the distal femur with extensive comminution after an accident car (Fig. 1). After 3 days, the patient underwent an open reduction and internal fixation of the fracture with irrigation and debridement at another hospital (Fig. 2). Post-operatively the patient underwent assisted continuous passive motion and muscular strengthening for 6 months (4 h per day). Weight bearing was allowed 3 months after surgery.

Seven months postoperatively the patient was able to walk with crutches and a knee brace with intensive knee pain. Range of motion of the knee (extension/flexion) was 0-40°. Radiographs of the right knee showed a delayed union of the right supra-intercondylar distal femoral fracture. The patient underwent a knee arthroscopy to perform a release of the adhesions and to gain range of motion. Post-operatively the patient underwent assisted continuous passive motion and muscular strengthening for a 5-month period. Weight bearing was allowed the day after the procedure. Range of motion after 5 months of exhausting physiotherapy was 0-60 ° (extension/flexion).

Despite of continuous physiotherapy, at 2 year follow up, the patient was not able to walk without crutches and knee brace, and he had intensive knee pain.

**Fig. 3:**
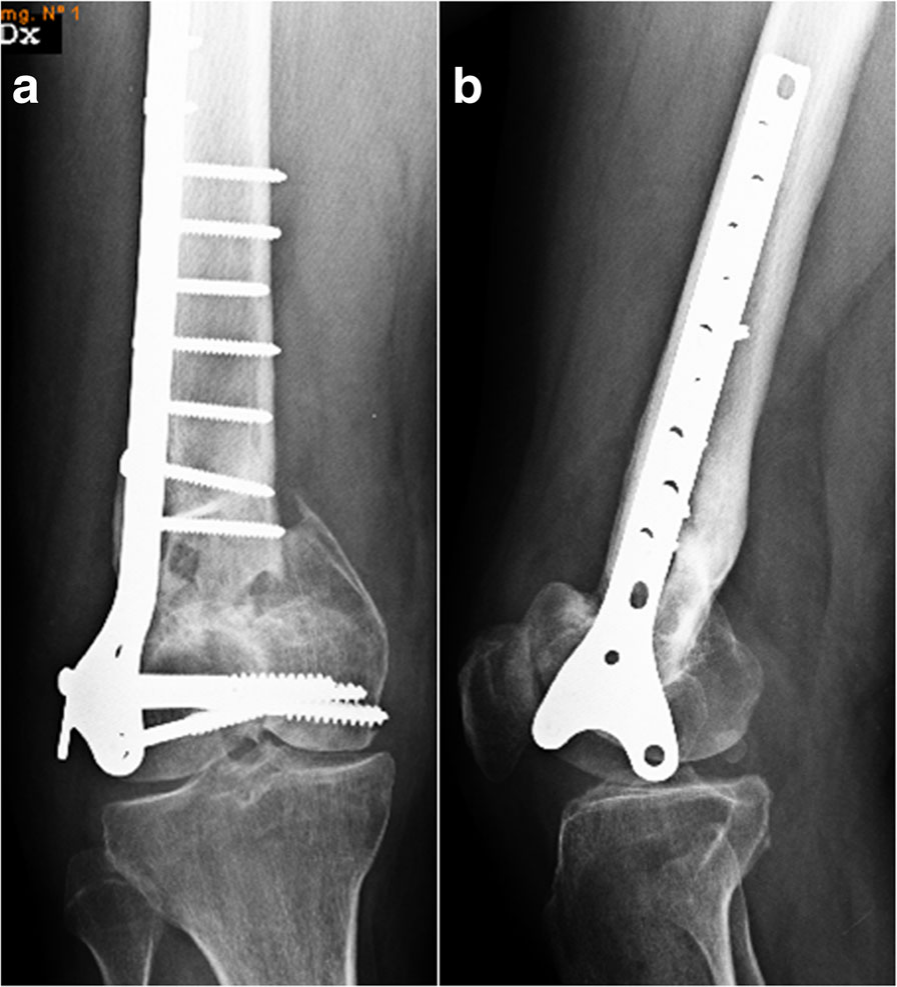
**a** Anteroposterior radiograph pre-operative of hardware removal; **b** Lateral radiograph pre-operative of hardware removal

Radiographs of the right knee showed a non complete union of the right supra-intercondylar distal femoral fracture and a varus deformity of the femur (Fig. 3). The patient underwent a labeled leukocyte imaging showing increased uptake in correspondence of the distal femur. Laboratory tests were normal. The infected non-union was classified in type A1 according to Jain AK et al. [5].

The patient underwent hardware removal and bone allograft implantation. Samples for microorganism cultures were obtained at surgery from surgical wound, non-union fibrous tissue, curetted bone and canal marrow cavity, but no pathogens were identified. Culture was performed also on the bone allograft, but no pathogens were identified. After surgery, the patient started antibiotic therapy for the management of the suspected osteomyelitis with teicoplanin 800 mg/die i.v. and levofloxacin 750 mg/die per os.

**Fig. 4:**
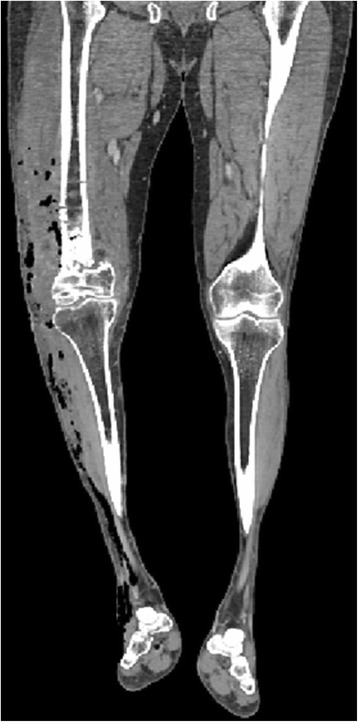
A CT scan showed the subcutaneous emphysema of the leg

**Fig. 5:**
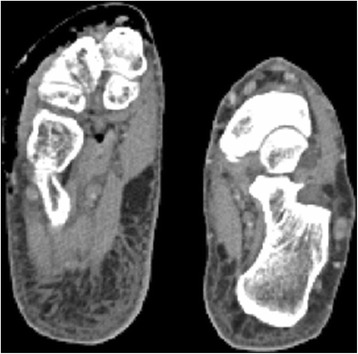
A CT scan showed extension of the emphysema to the toes

24 h postoperatively, the patient developed emphysema of the right lower limb. A CT scan showed the subcutaneous emphysema of the leg (Fig. 4), with extension to the toes (Fig. 5). The patient underwent exploration and debridement of the surgical wound, and multiple fasciotomies. Samples from surgical wound and soft tissues for microorganism cultures were obtained at surgery, but no pathogens were identified. Post-operatively a VAC (vacuum-assisted closure) system was applied. The patient underwent maximum dosage of broad spectrum antibiotic therapy for 14 days, consisting of ampicillin 18 g/die i.v., clindamicin 2.4 g/die i.v. and teicoplanin 800 mg/die i.v. Because increased values of the hepatic enzymes were observed after 3 days, teicoplanin was substituted with vancomycin 2 g/ die i.v.. The patient had complete resolution of subcutaneous emphysema of the leg in the postoperative period. 14 days postoperatively the VAC system was removed, and surgical wounds were closed.

The patient underwent a progressive rehabilitation programme. Weight bearing was allowed since the first day after removal of the VAC. The patient started again a continuous passive motion for 4 months post-operatively. The range of motion was 0°-60°. The patient underwent antibiotic therapy with levofloxacin 500 mg/die and rifampicin 900 mg/die for 3 months.

**Fig. 6:**
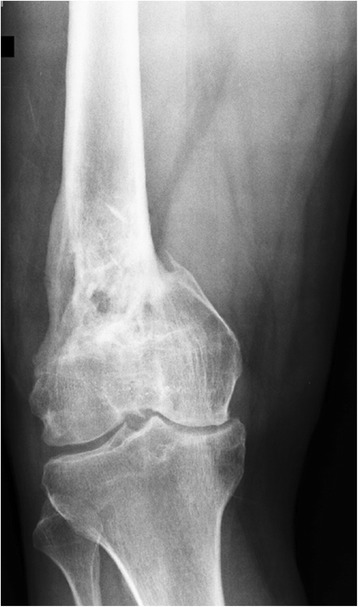
Anteroposterior radiograph of the right femur 2 year after the subcutaneous emphysema of the leg

At 4 year follow-up, the patient underwent radiographs (Fig. 6) and a labeled leukocyte imaging showing absence of uptake in correspondence of the distal femur. The patient’s limb was saved.

## Discussion

Hardware removal is a common orthopaedic procedure and it is frequently performed for symptoms attributed to the presence of hardware and in case of implant failure, infection, non-union, and soft-tissue compromise. As any surgical procedure, hardware removal carries inherent risks, including wound complications, iatrogenic injury, and anaesthetic complications [8]. The most common complication of hardware removal is infection, although gas gangrene has not been reported to date as a complication of hardware removal [1, 2].

Gas gangrene is a life-threatening disease. A traumatic injury, especially outdoor, exposes people to ground or other organic staff that could contain *C. perfringens* or other bacteria. In this way bacteria are introduced into soft tissues [9]. Healthy oxygenated tissues are hostile environments for the grow of bacteria. However a damage to blood supply suppresses the inflammatory response and allows the uncontrolled colonization of bacteria: the result could be a fulminant gas gangrene. The infection expands to other tissues, so the whole limb could become gangrenous in few hours making the amputation of the limb or removal of all of the infected tissues the only therapeutic strategies. Without this, the toxins produced by the bacteria rapidly kills the patient [9].

Although nonclostridial gas gangrene is not an unusual occurrence, relatively few cases have been reported. Due to the serious nature of some of these infections, it is important for physicians to familiarize themselves with these nonclostridial crepitant infections, which are often confused with clostridial myonecrosis [10]. Unfortunately, despite of our efforts, we were not able to determine the nature of the gangrene (clostridial or non nonclostridial). Multiple samples were performed both at surgery for hardware removal and during fasciotomies, as well as hemoculture and culture of the allograft, but no pathogens were isolated. The results of the cultures were probably a false negative [11]. Otherwise, we have not a scientific explanation to the emphysema (all the way down to the toes of the foot) and for the increased uptake at the bone scan. In clinical practice, sometimes, despite clinical diagnosis of infection, hemoculture and culture are negative and it is not possible to isolate pathogens responsible of the disease. In these cases, it is important to apply general principal and utilize large spectrum antibiotics. This strategy allowed us to save the limb in this patient.

Primary wound closure remains controversial in these patients, because of concerns for gas gangrene. Partial wound closure is an alternative, with delayed wound closure within 3 to 7 days. The use of sub-atmospheric pressure dressings, available commercially as the VAC device can be a valid alternative option in selected patients [12]. Wounds heal better with a sub-atmospheric pressure of 125 mmHg, especially if a pressure cycle of 5 min of suction alternated by 2 min off suction is utilized. In this way the blood flow is optimized, local tissue edema decreases, and the excessive liquid from the wound bed is removed. Moreover, the cyclic use of sub-atmospheric pressure changes the cytoskeleton of the cells in the wound bed and activates a cascade of intracellular signals leading to cell division and formation of granulation tissue. The combination of these mechanisms makes the VAC device a useful instrument for orthopaedic surgeon. Before surgery for hardware removal, we performed a review of the literature, to establish whether to perform a one- or a two- stage procedure: the optimal operative management of infected nonunions was not clear. Union rate for one stage strategies is between 70 and 100% and persistent infection percentages is between 0 and 55%. Instead for two-stage strategies union rate is between 66 and 100% and persistent infection percentages is between 0 and 60%. [1]. Because of patient requests and our experience in the field, and because of the low level of evidences (at best level IV, and suggesting similar results), we decided to perform a one-stage procedure with bone allografting. Even allografts are considered sterile, clostridium infections have been described after bone allograft implantation [13–17].

After the death from Clostridium sordellii sepsis of a 23-year-old man who had received a cadaveric musculoskeletal allograft [14], the Centers for Disease Control and Prevention (CDC) monitored the cases of allograft-associated clostridium infections and investigated processing and testing methods used by the tissue bank Recommendations have been made to tissue banks, the American Association of Tissue Banks, and the FDA to reduce the risk of allograft-associated infection to use a validated sporicidal process to confer the greatest protection for patients [14]. The bone allograft we used in our patient underwent the guidelines for allograft preparation. Therefore, we are not able to understand whether the infection arises from the bone allograft or from the site of infected non-union of the right femur of our patient.

## Conclusion

Although subcutaneous emphysema of the leg after hardware removal and bone allografting for infected non-union of the distal femur is extremely rare, the potential life treating complications and their potential impact on the functional status of the patient have to be taken into consideration when counseling patients about this procedure. Even when it is not possible to identify a bacterial pathogen responsible for the subcutaneous emphysema of the leg, prompt intervention may save the limb of the patient.

## Abbreviations

*C. perfringens*: *Clostridium perfringens*
CDC: Centers for disease control and prevention
CT: Computed tomography
VAC: Vacuum-assisted closure
